# The Antidepressant Mirtazapine Inhibits Hepatic Innate Immune Networks to Attenuate Immune-Mediated Liver Injury in Mice

**DOI:** 10.3389/fimmu.2019.00803

**Published:** 2019-04-12

**Authors:** Wagdi Almishri, Abdel Aziz Shaheen, Keith A. Sharkey, Mark G. Swain

**Affiliations:** ^1^Liver Unit, Snyder Institute for Chronic Disease, University of Calgary, Calgary, AB, Canada; ^2^Cumming School of Medicine, Hotchkiss Brain Institute, University of Calgary, Calgary, AB, Canada

**Keywords:** cytokine, chemokine, autoimmunity, macrophage, neutrophil, flow cytometry, inflammation

## Abstract

Activation of the innate immune system, including tissue macrophages and associated neutrophil infiltration, is an important driver of subsequent adaptive immune responses in many autoimmune diseases, including autoimmune hepatitis (AIH). The antidepressant mirtazapine has a unique complex pharmacology, altering signaling through a number of serotonin and histamine receptors that can impact macrophage function; an effect potentially influencing AIH outcome. In the mouse model of concanavalin A (Con A) induced liver injury (mimics many aspects of human AIH), in which early innate immune activation (i.e., stimulated hepatic macrophages/monocytes recruit neutrophils and additional monocytes to the liver) critically drives immune-mediated hepatitis induction, mirtazapine strikingly and dose-dependently inhibited Con A-induced liver injury. This inflammation-suppressing effect of mirtazapine was linked to an attenuation of Con A-stimulated early innate immune responses within the liver, including inhibition of hepatic macrophage/monocyte activation, decreased hepatic macrophage/monocyte-derived pro-inflammatory cytokine (e.g., TNFα) and chemokine (e.g., CXCL1 and CXCL2) production, suppression of Con A-induced increases in the hepatic expression of the neutrophil relevant endothelial cell adhesion molecule ICAM-1, with the resultant significant reduction in neutrophil recruitment into the liver. Consistent with our findings in the Con A model, mirtazapine also significantly reduced activation-induced release of cytokine/chemokine mediators from human CD14^+^ monocytes *in vitro*.

**Conclusion:** Our data suggest that mirtazapine can attenuate hepatic innate immune responses that critically regulate the subsequent development of autoimmune liver injury. Therefore, given that it is a safe and widely used medication, mirtazapine may represent a novel therapeutic approach to autoimmune liver disease.

## Introduction

Classically, autoimmune disease was considered a disorder of adaptive immunity (1). However, early innate immune responses are clearly important for driving subsequent adaptive immune responses in autoimmunity. In numerous autoimmune disease models, activation of resident tissue macrophages, monocytes, and neutrophil recruitment critically regulate tissue inflammation and contribute to autoimmune-mediated tissue injury (2–4). In autoimmune liver disease, enhanced hepatic recruitment and activation of macrophages, monocytes and neutrophils is commonly observed, but the importance of these innate immune cell types in regulating the autoimmune process has not been broadly appreciated (5, 6). Interestingly, the majority of patients with type I AIH develop anti-neutrophil cytoplasmic antibodies (ANCA), suggesting the involvement of neutrophils in regulating liver autoimmunity in these patients (7). In addition, patients with AIH exhibit altered macrophage function (8). Therefore, alterations in hepatic innate immunity may play an important role in regulating the development of AIH.

In chronic medical conditions the prevalence of depressive symptoms is high, often resulting in prescription of antidepressants (9). However, in addition to their effects on symptoms, antidepressants can alter immunity in animal models and patients, including impacting innate immune responses (10, 11). Mirtazapine is an atypical antidepressant with complex pharmacology, including antagonist activity at multiple receptor subtypes including norepinephrine (α_2_ adrenergic), serotonin (5HT; 5HT2_a_, 5HT3) and histamine (H1) receptors, and antagonist/inverse agonist activity at the 5HT2_c_ receptor (12). Given these broad range of receptor interactions, mirtazapine has been widely employed clinically to treat depression and other symptoms including anorexia, poor sleep and anxiety (13). Importantly, both serotonin and histamine clearly modulate immunity (14–16). Furthermore, mirtazapine-active receptors are expressed on macrophages/monocytes and can alter their function (14–17). Therefore, it is plausible that mirtazapine treatment may impact hepatic immunity, with associated effects on liver autoimmunity. Consistent with this, we recently identified that mirtazapine treatment (uniquely amongst all antidepressant classes) improves hepatic outcomes and survival in patients with the autoimmune liver disease primary biliary cholangitis (PBC) (18).

Concanavalin A (Con A)-induced liver injury is a well-established mouse model of immune-mediated liver injury, resembling many aspects of human AIH (19, 20). Con A treatment induces a cascade of immunological events within the liver, including early innate immune responses characterized by increased hepatic macrophage/monocyte activation and TNFα production, which is a critical driver of liver injury (19, 21). TNFα induces the expression of the adhesion molecule ICAM-1 on hepatic sinusoidal endothelium (22), and increased hepatic production of the important macrophage-derived neutrophil chemokines CXCL1 and CXCL2, which together rapidly recruit neutrophils into the liver (23, 24). We have previously shown that this early hepatic recruitment of neutrophils is critically important for driving Con A-mediated liver injury (25).

Therefore, we undertook this series of experiments to delineate the impact of mirtazapine on immune-mediated liver injury in the Con A model, and determine the mechanism whereby mirtazapine treatment impacts Con A-induced liver injury; possibly by altering early innate immune responses within the liver that are activated after Con A treatment.

## Methods

### Con A-Induced Immune Mediated Liver Injury Model

Male 8–10 week old C57BL/6 mice (Jackson Labs, Bar Harbor, Maine) were used. All procedures were approved by the University of Calgary Animal Care Committee (protocol numbers AC14-0129, AC14-0128) and were performed in accordance with the guidelines of the Canadian Council on Animal Care. The Con A hepatitis model is a widely used and well-characterized model of immune-mediated liver injury that mimics many aspects of AIH in patients (19, 20). Mice were treated with Con A (13.5 mg/kg iv; Sigma, St. Louis, MO) or vehicle (PBS) (26), and were sacrificed 16 h later. Hepatitis severity was determined biochemically (ALT) and histologically (H & E staining) (26). A second mouse model of immune-mediated liver injury, resulting from the administration of alpha galactosylceramide (αGalCer), was used to assess the impact of mirtazapine treatment on hepatitis severity. In this well-characterized model a single intraperitoneal injection of αGalCer (100 μg/kg; Cayman Chemical, Ann Arbor, Michigan, USA) induces a moderate hepatitis (maximal at 16 h post-αGalCer administration) that is independent of hepatic macrophage/monocyte activation (27, 28). All experiments were repeated at least twice unless otherwise noted.

### Antibodies and Other Reagents

The following reagents, antibodies (and appropriate isotype controls) were obtained from indicated sources: Percoll® (GE HealthCare Biosciences, Baue D'urfe, Quebec, Canada), Naphthol AS-D Chloroacetate (Specific Esterase) Kit, Hematoxylin solution, Gill No. 3, protease inhibitor cocktail, anti-mouse CD16/CD32 (93), RPMI 1,640 medium, HEPES, fetal bovine serum (FBS), UltraPure™ DNase/RNase-Free Distilled Water and phosphate-buffered saline (PBS), Non-Essential Amino Acids Solution (100X), L-glutamine (200 mM), sodium pyruvate (100 mM), penicillin-streptomycin (10,000 U/mL) (Thermo Fisher Scientific, MA, USA ). Anti-mouse CD11b (M1/70), rat anti-mouse Ly6G (1A8), anti-mouse CD45 (30-F11), anti-mouse CD45 (30-F11), anti-mouse F4/80 (BM8), anti-mouse CD3ε (145-2C11), anti-mouse CD45 (30-F11), anti-mouse CD80 (16-10A1), anti-mouse CD4 (RM4-4), (BioLegend, CA, U.S.A). Anti-mouse Ly6C (HK1.4), anti-mouse MHC Class II (M5/114.15.2), anti-mouse IFNγ (XMG1.2), anti-mouse CD69 (H1.2F3), anti-mouse TNFα (TN3-19.12), anti-mouse Ly6C (HK1.4) from eBioscience (San Diego, CA, USA). Mirtazapine (CAS No: 85650-52-8; Tocris Bioscience, Bristol, UK) and TWEEN^®^ 80 (Proteomics grade CAS Number: 9005-65-6; Amresco LLC, OH, USA). Anti-mouse ICAM-1 antibody (YN1/1.7.4) and antigen retrieval buffers (100X EDTA buffer, pH 8.0 and 100x Citrate buffer pH 6.0) (Abcam, Cambridge, UK). Serotonin (ab133053) and histamine ELISA kits (ab213975) from Abcam (Cambridge, UK). Avidin/Biotin Blocking Kit, ImmPACT NovaRED Peroxidase (HRP) substrate, Vecstain Elite ABC HRP Kit (Peroxidase, Standard), biotinylated goat anti-rat IgG antibody, permanent non-aqueous mounting medium, normal goat serum (Vector Laboratories Inc., CA, USA). Microtainer diagnostic K2 EDTA tubes (BD Biosciences, San Jose, CA, USA). Human CD14^+^ positive selection kit (Mylteni Biotec Bergisch, Gladbach, Germany). BCA Protein Assay kit (23227) (Pierce, USA).

### Mirtazapine Treatment and Con A Hepatitis Severity

To delineate the impact of mirtazapine treatment in Con A hepatitis, mice were treated 1 h prior to Con A treatment with mirtazapine 1–20 mg/kg intraperitoneally (ip) (29). Blood and liver samples were collected under isoflurane anesthesia 16 h post-Con A treatment (unless otherwise noted) to assess liver injury biochemically (plasma alanine aminotransferase [ALT] activity; measured using Roche-Hitachi Modular-P800 apparatus; Roche, Mannheim, Germany) and histologically using formalin-fixed liver tissue slices stained with Hematoxylin and Eosin (H&E) (26, 28). Extent of liver parenchymal necrosis was quantitated as previously described using Image J software and an Olympus XC10 camera (acquired using the Olympus VS-ASW software package; original magnification x400) (28). In additional experiments, mirtazapine (20 mg/kg ip) was administered 2 h *after* Con A treatment (i.e., therapeutically) and mice sacrificed 16 h later and severity of liver injury determined by ALT measurement.

In further experiments, the impact of specifically blocking individual receptors known to be impacted by mirtazapine treatment (i.e., 5HT2a, 5HT2c, 5HT3, and H1; also 5HT1a receptor) (13, 30) on the severity of Con A hepatitis was determined by ALT measurement. Receptor antagonists examined include sarpogrelate hydrochloride (selective 5HT2a antagonist), granisetron hydrochloride (5HT3 antagonist), S 32212 hydrochloride (5HT2c inverse agonist; α2 antagonist), (S)-WAY 100135 dihydrochloride (selective 5-HT1a receptor antagonist) (Tocris Bio-Techne). Cetirizine dihydrochloride (Histamine one receptor) (Sigma-Aldrich Canada Co., Oakville, Ontario). All drugs were administered ip (10 mg/kg) except cetirizine dihydrochloride, which was given by oral gavage. Animals were divided into groups that received either Con A alone (13.5 mg/kg) or Con A + receptor antagonist. Sixteen hours post-Con A injection plasma samples were collected ALT levels measured.

### Impact of Mirtazapine Treatment on Con A-Induced Hepatic Macrophage/Monocyte Activation

The liver contains a sessile, self-renewing population of fixed tissue macrophages called Kupffer cells (F4/80^+^). In addition, the normal liver contains two main populations of tissue resident monocytes, an “inflammatory” subgroup (Ly6C^hi^ CCR2^hi^) which patrol the hepatic sinusoids, and a less abundant tissue “repair” subgroup (Ly6C^int/lo^CX3CR1^hi^) (31, 32). During inflammation, macrophages, and monocytes are rapidly activated, secrete a number of cytokines (including TNFα), and express cell surface MHC II and co-activating signals such as CD80 which are important for regulating subsequent adaptive immune responses (31–37). In addition, recruitment of monocytes to the liver is enhanced and the dynamic activation of these cells critically regulates liver injury and repair processes (33, 38). Therefore, to determine the impact of mirtazapine on Con A-induced hepatic macrophage/monocyte activation, hepatic mononuclear cells were isolated using Percoll® at 3 and 16 h post-Con A treatment, and subjected to direct immunofluorescence analyses using flow cytometry, as previously described (26, 28). Samples were acquired using either a FACScan flow cytometer (Becton Dickinson, Mountain View, CA), or Attune™ Acoustic Focusing flow cytometer (Applied Biosytems, Mainway, Burlington, ON). Data was analyzed using FlowJo® software (Treestar, Ashland, OR). Gating proceeded as follows: gating of live cells and exclusion of duplet cells, followed by gating on CD45^+^ leukocytes. Within the CD45^+^ leukocyte gate, macrophages were identified as CD11b^lo/neg^Ly6C^neg^F4/80^+^ cells (Kupffer cells; KCs), and monocytes (CD11b^+^Ly6G^−^ Ly6C^+^ cells) were identified and subdivided into two groups based on gating: (i) “inflammatory” monocytes (IMs; Ly6C^hi^), and “repair” monocytes (RMs; Ly6C^int/low^) (39). In addition, activation of hepatic macrophages/monocytes was determined by cell surface expression of MHC II and CD80, and by the cellular production of the cytokine TNFα which is a central regulator of Con A hepatitis (40) and AIH severity in patients (41). Fluorescence-minus-one (FMO) controls accurately identified cells with fluorescence above background (28). Appropriate isotype controls determined antibody specificity.

### Mirtazapine–Mediated Alterations in Con A-Induced Hepatic Recruitment of Neutrophils and CD4 T Cells to the Liver

We have previously shown that neutrophil recruitment critically regulates Con A hepatitis (25), and activation of liver macrophages/monocytes plays a key role in this process (19, 24). Therefore, we determined the impact of mirtazapine treatment on Con A-induced hepatic recruitment of neutrophils in paraffin-embedded liver sections 16 h post-Con A treatment, using esterase (25) and Ly6G staining (42). Hepatic leukocyte esterase expression was identified using a naphthol AS-D-chloroacetate esterase staining kit, according to the manufacturer's protocol, and Ly6G^+^ cells identified using immunohistochemistry. Briefly, following tissue deparaffinization and rehydration antigen retrieval was performed in 1X citrate buffer (pH 6.0; 95–100°C for 20 min). Endogenous peroxidase and endogenous biotin binding were blocked using 3% H_2_O_2_ and an avidin/biotin blocking kit (Vector Laboratories, Burlingame, CA), respectively. Slides were incubated with rat primary anti-mouse Ly6G monoclonal antibody (1 μg/ml overnight at 4°C) followed by incubation with biotinylated goat anti-rat secondary antibody (1:150 dilution. 1 h at room temperature; Vector Labs). Staining controls were performed by omission of primary antibody, or of both primary and secondary antibodies. In all cases, negative controls showed no significant staining. Slides were incubated with Vectastain Elite ABC kit (Vector Laboratories) for 30 min at room temperature. Color was developed with Nova Red Chromogen (ImmPACT NovaRED Peroxidase Substrate, Vector Lab, Burlingame, CA), and counterstained with hematoxylin (EMDmillipore). Mounted slides were converted to virtual slides with a BX 61 VS virtual microscopy system equipped with an XC10 camera and VS ASW software (Olympus; original magnification 400X). All virtual liver sections were examined in a blinded fashion and esterase^+^ and Ly6G^+^ cells counted and reported as number of positive staining cells/HPF (25).

During Con A hepatitis neutrophils are mobilized from the bone marrow into peripheral blood and then recruited to the liver (43). Therefore, we measured the impact of mirtazapine treatment on circulating neutrophil numbers within inferior vena cava blood samples (BD microtainer diagnostic K2 EDTA tubes) 8 h post-Con A treatment. The absolute neutrophil count was determined using an automated Coulter full blood counter and expressed as cells/L (Calgary Lab Services, Calgary, Canada) (25, 43).

CD4^+^ T lymphocytes are important adaptive immune effector cells in the development of Con A-induced liver injury, as blocking T cell function prevents Con A hepatitis (44). Similarly, CD4 T cells are critical regulators of liver injury and progression in AIH patients (45). Therefore, in additional experiments we determined the impact of mirtazapine treatment on subsequent CD4 T cell recruitment and activation within the liver 16 h post-Con A. Hepatic CD4 T cells were isolated using Percoll®, subjected to direct immunofluorescence analyses using flow cytometry, and identified as CD45^+^CD3^+^CD4^+^ cells. T cell activation was measured by cellular expression of the activation marker CD69, and by production of the cytokine IFNγ (by flow cytometry) (28).

### Impact of Mirtazapine Treatment on Con A-Induced Increases in Hepatic Levels of Neutrophil Recruitment-Relevant Macrophage-Derived Cytokines and Chemokines, and Upregulation of Hepatic ICAM-1 Expression

Activated hepatic macrophages/monocytes are key regulators of neutrophil recruitment into the liver during Con A hepatitis, through production of the cytokines TNFα and IL-6, the chemokines CXCL1 and CXCL2, and through the TNFα-mediated upregulation of the neutrophil endothelial adhesion molecule ICAM-1 (19–24, 43). Therefore, we determined the impact of mirtazapine treatment on the Con A-induced increases in hepatic expression of these mediators.

#### Hepatic Cytokine/Chemokine Levels

Hepatic levels of the cytokines TNFα and IL-6, and neutrophil-relevant chemokines CXCL1 and CXCL2, were measured in liver homogenates by Luminex® (Eve Technologies Corporation, Calgary, Canada) (28). Liver homogenate protein concentrations were quantified using a BCA Protein Assay kit (Pierce, USA). Results expressed as pg/mg protein.

#### Liver Expression of the Adhesion Molecule ICAM-1

Con A treatment robustly increases hepatic ICAM-1 expression, mainly in sinusoidal endothelium (22). HepaticICAM-1 expression was determined using immunohistochemistry. Briefly, following tissue deparaffinization and rehydration antigen retrieval was performed in 1X EDTA buffer pH 8.0 (95–100°C, 20 min). Endogenous peroxidase and endogenous biotin binding were blocked using 3% H_2_O_2_ and an avidin/biotin blocking kit (Vector Laboratories, Burlingame, CA), respectively, and slides incubated with rat primary anti-mouse ICAM-1 monoclonal antibody (clone YN1/1.7.4; 1:200 dilution, overnight at 4°C), followed by incubation with biotinylated goat anti-rat secondary antibody (1:150 dilution) for 1 h at room temperature (Vector Labs). The remaining steps are identical to those reported for Ly6G staining above.

### Impact of Mirtazapine on Hepatic Serotonin and Histamine Levels in Con A Hepatitis

Livers were flushed with ice cold saline and removed from mice 6 h post-Con A (or vehicle), which had been treated with mirtazapine or vehicle (46). Hepatic serotonin and histamine levels were then determined by ELISA (following manufacturers guidelines).

### Mirtazapine Effects on Cytokine/Chemokine Production by Human Monocytes and CD4 T Cells *in vitro*

CD14^+^ monocytes were isolated from healthy donor peripheral blood using an autoMACS Separator and autoMACS CD14^+^ positive selection kit (Mylteni Biotec Bergisch, Gladbach, Germany). CD14^+^ cells were seeded into 24-well tissue culture plates (density of 1 × 10^6^ cells/well) in 500 μl RPMI 1,640 medium supplemented with 10% FBS, 1 mM sodium pyruvate, 2 mM L-glutamine, and 100 units/ml penicillin and streptomycin, and non-essential amino acids (NEAA). After 4 h incubation (5% CO_2_, 37°C) non-adherent cells were removed by washing, and 500 μl of pre-warmed complete fresh media added to wells. Designated wells were treated with mirtazapine (10 μM) or vehicle (0.2 μl/ml DMSO). One hour later Con A (5 μg/ml) or vehicle were added to designated wells, and cells cultured for another 24 h (47). Supernatants were collected and stored at −80°C until assayed for cytokine/chemokine levels (expressed as pg/ml).

CD4^+^ T cells were isolated from healthy donor peripheral blood using EasySep™ Human CD4^+^ T cell isolation kit (STEMCELL Technologies Canada Inc, Vancouver, BC). Purity of isolated cells as tested by flow cytometry was >97%. Cells were cultured in a 24-well plate (density 10^6^ cells/well) in 500 μl RPMI 1,640 medium supplemented with 10% FBS, 1 mM sodium pyruvate, 2 mM L-glutamine, and 100 units/ml penicillin and streptomycin, and non-essential amino acids (NEAA). Designated wells were treated with mirtazapine (10 μM) or vehicle (0.2 μl/ml DMSO). One hour later Con A (5 μg/ml) or vehicle were added to designated wells, and cells cultured for another 24 h. Supernatants were collected and stored at −80°C until assayed for cytokine levels. Human IL-10, IL-4, and IFNγ were measured in culture supernatants using a human MILLIPLEX kit (Millipore, USA) according to the manufacturer's protocol. The multiplexing analysis was performed using the Luminex 100 system (Luminex®, USA) (Eve Technologies Corporation, Calgary, Canada).

### Statistical Analysis

All data shown as mean ± standard error of the mean (SEM). For comparisons between two groups, an unpaired Student's *t*-test was used. For comparisons between more than two groups an analysis of variance followed by the Student-Newman-Keuls *post-hoc* test was performed (Graph-Pad V5, San Diego, CA). A *p*-value of ≤0.05 was considered significant.

## Results

### Mirtazapine Treatment Significantly Attenuates Con A-Induced Hepatitis

**Figure 1:**
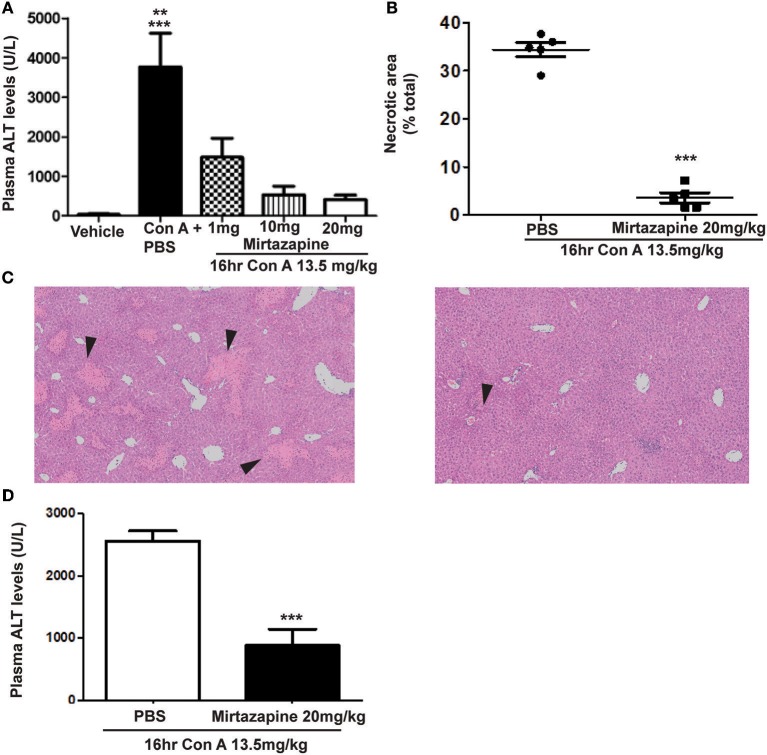
Mirtazapine pretreatment attenuates Con A-induced immune-mediated hepatitis. **(A)** Administration of mirtazapine leads to a marked reduction in liver damage 16 h post-Con A treatment, as reflected by plasma ALT levels (^***^p < 0.001 Con A + PBS group vs. vehicle, Con A + 10 mg/kg mirtazapine group, and Con A + 20 mg/kg mirtazapine groups; ^**^p < 0.01 Con A + PBS group vs. Con A + 1 mg/kg mirtazapine group; *n* = 4–5 mice/group), and by **(B)** quantification of histological damage in H&E stained liver sections (i.e., as total (%) area of liver cell necrosis). **(C)** Representative H&E stained liver sections from Con A-treated mice that received either mirtazapine (20 mg/kg) (**C**, right panel) or mirtazapine vehicle (**C**, left panel). Mice treated with Con A + vehicle showed extensive liver cell necrosis (black arrowheads) whereas mice treated with Con A + mirtazapine showed only minimal hepatocyte damage (images are 100 X). **(D)** Mirtazapine treatment 2 h after Con A administration significantly attenuates Con A hepatitis 16 h post-treatment. ^***^p < 0.0007; *n* = 4 (Con A) and 5 (Con A + mirt) mice per group.

As previously reported, Con A treatment resulted in a robust elevation in plasma ALT levels (Figure 1A) (25, 26), and mirtazapine treatment dose-dependently reduced these Con A-induced increases (Figure 1A). Mirtazapine-induced improvements in plasma ALT levels were paralleled by a striking reduction in hepatocyte necrosis histologically (Figures 1B,C). Based on dose response experiments (Figure 1A), the 20 mg/kg dose was selected for further experiments in the Con A model. In addition, mirtazapine given therapeutically (i.e., 2 h post-Con A treatment) attenuated Con A-induced liver injury as reflected by a significant improvement in serum ALT levels (Figure 1D). Moreover, in the model of αGalCer induced immune-mediated hepatitis, in which liver injury is independent of hepatic macrophages/ monocytes, mirtazapine treatment did not alter hepatitis severity as reflected by ALT levels (ALT in U/L; vehicle: 19.0 ± 1.1 vs. αGalCer + vehicle: ^*^251.4 ± 29.8 vs. αGalCer + mirtazapine: ^*^271.0 ± 61.7; ^*^p < 0.01 vs. vehicle alone group; *n* = 5 mice per group).

### Inhibition of Con A-Induced Hepatic Monocyte/Macrophage Activation by Mirtazapine Treatment

**Figure 2:**
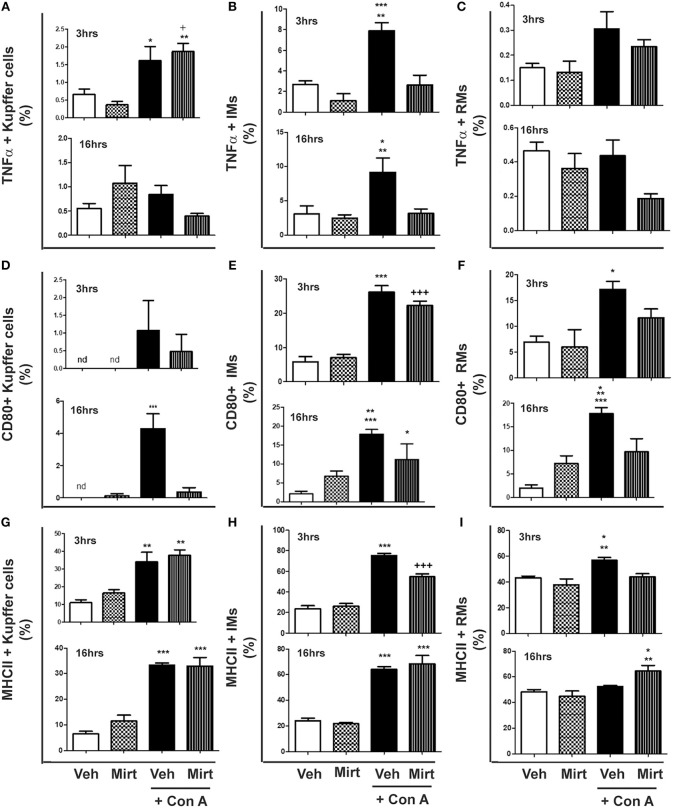
Mirtazapine treatment inhibits Con A-induced hepatic monocyte/macrophage activation. Mice were treated with vehicle (veh), mirtazapine (mirt; 20 mg/kg), Con A + veh or Con A + mirt. At 3 and 16 h post-Con A treatment hepatic immune cells were isolated and the effect of mirt treatment on hepatic monocyte/macrophage activation assessed by flow cytometry. **(A)** TNFα production by hepatic Kupffer cells; 3 h ^*^p < 0.05 Con A + veh vs. veh and mirt groups. ^+^*p* < 0.05 Con A + mirt vs. veh group. ^**^p < 0.01 Con A + mirt vs. mirt group. Sixteen hours groups were not statistically different. **(B)** TNFα production by hepatic IMs; 3 h ^***^p < 0.0001 Con A + veh vs. veh and mirt groups. ^**^p < 0.01 Con A + veh vs. Con A + mirt group; 16 h ^*^p < 0.05 Con A + veh vs. veh and mirt groups. ^**^p < 0.01 Con A + veh vs. Con A + mirt group. **(C)** TNFα production of hepatic RMs; 3 and 16 h, groups are not statistically different. **(D)** CD80 expression on hepatic KCs; 3 h, groups are not statistically different. Sixteen hours ^***^p < 0.0001 Con A+ veh vs. all other groups. **(E)** CD80 expression on hepatic IMs; 3 h ^***^p < 0.0001 Con A+ veh vs. veh and mirt groups. ^+++^*p* < 0.0001 Con A + mirt vs. veh and mirt groups. Sixteen hours, ^***^p < 0.0001 Con A+ veh vs. veh. ^**^p < 0.01 Con A+ veh vs. mirt. ^*^p < 0.05 Con A + mirt vs. Con A + veh and veh groups. **(F)** CD80 expression on hepatic RMs; 3 h, ^*^p < 0.05 Con A + veh vs. veh and mirt groups. Sixteen hours ^***^p < 0.0001 Con A+ veh vs. veh. ^**^p < 0.01 Con A+ vehicle vs. Con A + mirt. ^*^p < 0.05 Con A + veh vs. mirt. **(G)** MHC II expression on hepatic KCs; 3 h, ^**^p < 0.01 Con A+ veh and Con A+ mirt vs. veh and mirt groups. Sixteen hours, ^***^p < 0.0001 Con A+ veh and Con A+ mirt vs. veh and mirt groups. **(H)** MHC II expression on hepatic IMs; 3 h, ^***^p < 0.0001 Con A+ veh vs. all other groups. ^+++^*p* < 0.0001 Con A+ mirt vs. all other groups. Sixteen hours, ^***^p < 0.0001 Con A+ veh and Con A+ mirt vs. veh and mirt groups. **(I)** MHC II expression on hepatic RMs; 3 h, ^**^p < 0.01 Con A+ veh vs. veh. ^*^p < 0.05 Con A + veh vs. Con A + veh and mirt groups. Sixteen hours, ^**^p < 0.01 Con A+ mirt vs. mirt group. ^*^p < 0.05 Con A + mirt vs. Con A + veh and veh groups. *n* = 4–5 mice per group.

Con A treatment resulted in the differential activation of hepatic KC, IM, and RM populations, as reflected by TNFα production and cellular MHC II and CD80 expression:

(i) KCs: Con A treatment resulted in a low level but significant increase in the proportion of KCs producing TNFα at 3 h post-Con A, which was unaltered by mirtazapine treatment (Figure 2A). However, by 16 h post-Con A treatment the proportion of TNFα-producing KCs was similar to vehicle-treated mice for all groups (Figure 2A). In contrast, CD80 expression was barely detectable in KCs for all treatment groups at 3 h post-Con A (Figure 2D). However, at 16 h post-Con A treatment CD80 expression was significantly increased in KCs, and this Con A-induced increase was completely prevented by mirtazapine (Figure 2D). Con A treatment also resulted in a significant increase in MHC II expressing KCs 3 h post-Con A which persisted at 16 h post-Con A, but was not altered by mirtazapine (Figure 2G).

(ii) IMs: Con A treatment significantly increased IM production of TNFα at 3 and 16 h (Figure 2B), and mirtazapine completely suppressed the Con A-induced increase in TNFα production (Figure 2B). Con A also induced a significant increase in CD80 expressing IMs within 3 h that was unaltered by mirtazapine. This increase was sustained at 16 h after Con A treatment in the Con A group that did not receive mirtazapine (Figure 2E), but was significantly attenuated in IMs from Con A-treated mice that received mirtazapine (Figure 2E). In contrast to CD80, MHC II expressing IMs were increased at 3 h in both Con A-treated groups, compared to vehicle and mirtazapine alone groups, but this increase was significantly attenuated by mirtazapine treatment (Figure 2H). At 16 h post-Con A treatment, MHC II expressing IMs remained increased in the Con A treated groups and were similar in the Con A vs. Con A plus mirtazapine groups (Figure 2H).

(iii) RMs: Repair monocytes produced very low levels of TNFα at the 3 and 16 h time points, which was not altered by Con A or mirtazapine treatment (Figure 2C). CD80 expression in RMs was significantly increased by Con A treatment at 3 h compared to vehicle and mirtazapine alone groups, but not in the Con A plus mirtazapine group (Figure 2F). Increased RM expression of CD80 was sustained at 16 h post-Con A, but at the 16 h time point CD80 expression in the Con A plus mirtazapine group had returned to baseline levels (Figure 2F). MHC II expression in RMs was increased at 3 h post-Con A treatment, and this increase was completely prevented by mirtazapine treatment. However, by 16 h post-Con A MHC II expression in RMs had return to baseline, but was significantly increased in the Con A plus mirtazapine group compared to all the other groups (Figure 2I).

### Mirtazapine Treatment Significantly Reduces Con A-Induced Hepatic Neutrophil Recruitment

Hepatic macrophage activation after Con A treatment leads to production of cytokines (e.g., TNFα and IL-6) and chemokines (e.g., CXCL1 and CXCL2), in addition to TNFα-induced upregulation of the adhesion molecule ICAM-1, all of which critically regulate hepatic neutrophil recruitment (19, 21–25, 43). Importantly, hepatic neutrophil recruitment is essential for the subsequent development of Con A liver injury (25, 43, 48).

#### Mirtazapine Treatment Inhibits Neutrophil Recruitment to the Liver During Con A Hepatitis

**Figure 3:**
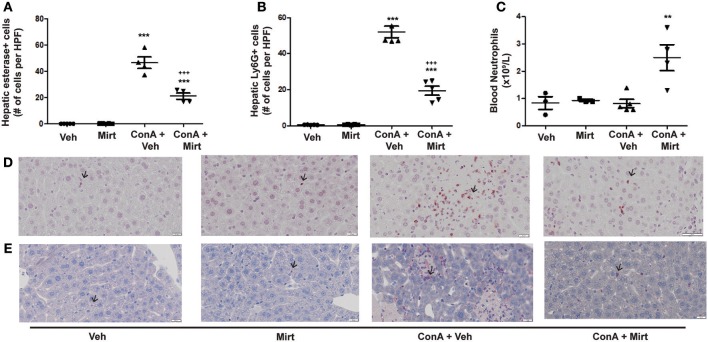
Mirtazapine treatment significantly decreases neutrophil recruitment into the liver, and increases circulating neutrophil numbers, in Con A treated mice. **(A,B)** Mirtazapine treatment significantly reduces Con A-mediated hepatic neutrophil recruitment, as reflected by esterase staining **(A)** or Ly6G staining **(B)**. Treatment with mirtazapine alone did not alter hepatic neutrophil numbers **(A,B)**. Administration of mirtazapine leads to a striking reduction in the number of neutrophils recruited to the liver post-Con A administration. Esterase or Ly6G positive cells were counted in 60 random high power fields (HPF)/liver section, and numbers of positive cells averaged for each liver section. Although mirtazapine treatment alone did not alter hepatic neutrophil numbers, administration of mirtazapine resulted in a >2-fold reduction in numbers of neutrophils recruited to the liver post-Con A administration. ^***^p < 0.0001 Con A + vehicle and Con A + mirt vs. mirt and vehicle alone groups. ^+++^*p* < 0.0001 vs. Con A + vehicle treated groups; *n* = 4–5 mice/group). **(C)** Significant increase in circulating blood neutrophil count in Con A + mirtazapine treated mice vs. Con A + vehicle, mirtazapine alone or vehicle alone treated groups, determined 8 h post-Con A treatment. ^**^p < 0.01 Con A + mirtazapine vs. other groups; *n* = 3–5 mice/group). **(D)** Representative immunohistochemical images of Ly6G^+^ staining neutrophils (black arrows) in liver sections. **(E)** Representative immunohistochemical images of positive esterase staining immune cells (black arrows) in liver sections.

Con A treatment rapidly recruits neutrophils into the liver (25), and mirtazapine treatment significantly attenuates the Con A-induced influx of neutrophils into the liver, as reflected by neutrophil staining with esterase (Figures 3A,E) (25) and Ly6G (Figures 3B,D) (42). Mirtazapine treatment alone did not alter hepatic neutrophil numbers compared to vehicle alone treated mice (Figures 3A,B,D,E). Consistent with a mirtazapine-related reduction in hepatic neutrophil recruitment post-Con A treatment, circulating numbers of neutrophils in peripheral blood increased in mice treated with Con A plus mirtazapine vs. Con A alone (Figure 3C).

#### Significant Reduction in Con A-Induced Increases in Hepatic Levels of Neutrophil Recruitment Relevant Cytokines and Chemokines by Mirtazapine

**Figure 4:**
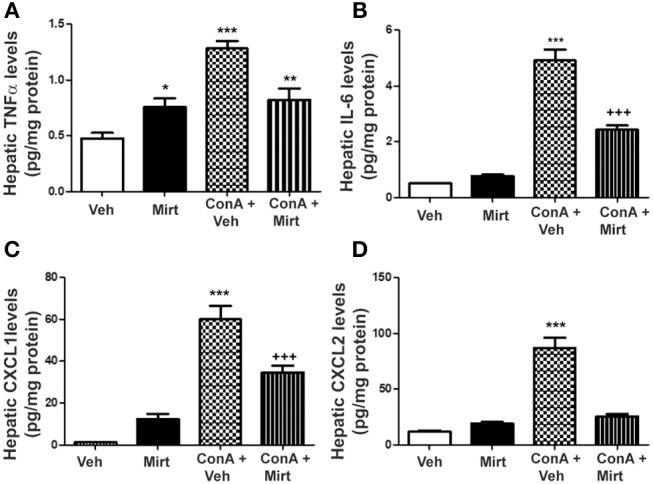
Mirtazapine administration significantly attenuates Con A-induced increases in hepatic cytokine (TNFα, IL-6) and chemokine (CXCL1, CXCL2) levels. **(A)** Mirtazapine treatment significantly increases hepatic TNFα levels, compared to vehicle treated animals, and Con A treatment further increases hepatic TNFα levels; an increase that is prevented by mirtazapine treatment. ^*^p < 0.05 mirtazapine vs. vehicle groups; ^**^p < 0.01 Con A + mirtazapine vs. vehicle group; ^***^p < 0.0001 Con A+ vehicle vs. all other groups; *n* = 6–7 mice/group). **(B)** Con A treatment significantly increases hepatic IL-6 levels which is attenuated by mirtazapine treatment. ^***^p < 0.0001 Con A + vehicle vs. all other groups; ^+++^*p* < 0.0001 Con A + mirtazapine vs. all other groups. *n* = 6–7 mice/group). **(C,D)** Con A treatment significantly increases hepatic CXCL1 levels, and mirtazapine treatment significantly attenuates this Con A-induced increase. ^***^p < 0.0001 Con A + vehicle vs. all other groups; ^+++^*p* < 0.0001 Con A + mirtazapine vs. all other groups. *n* = 6–7 mice/group. **(D)** Con A treatment significantly increases hepatic CXCL2 levels which is significantly attenuated by mirtazapine treatment. ^***^p < 0.0001 Con A + vehicle vs. all other groups. *n* = 6–7 mice/group.

Interestingly, mirtazapine treatment alone resulted in a significant increase in hepatic TNFα, but not IL-6, levels compared to vehicle-treated mice (Figures 4A,B). Hepatic macrophages/monocytes are the main hepatic TNFα producing cell type. Therefore, this finding suggests that mirtazapine may have a direct effect on TNFα production in these cells. As previously reported, Con A treatment significantly increases hepatic TNFα and IL-6 levels (Figures 4A,B) (19, 21, 22, 43), which were significantly attenuated by mirtazapine treatment (Figures 4A,B). Con A treatment also significantly increased hepatic levels of the neutrophil chemokines, CXCL1 and CXCL2 (Figures 4C,D), which was inhibited by mirtazapine treatment (Figures 4C,D).

#### Con A-Induced Increased Hepatic ICAM-1 Expression Is Markedly Reduced by Mirtazapine Treatment

**Figure 5:**
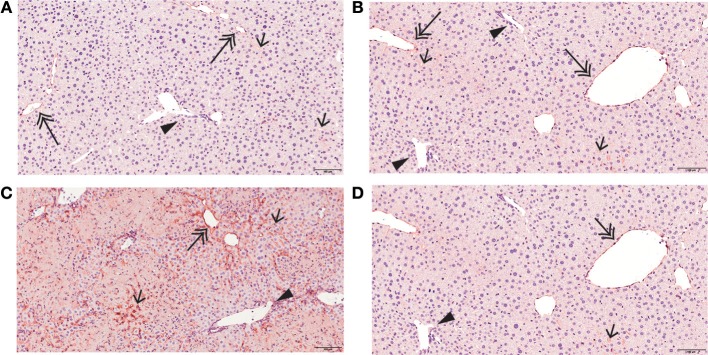
Mirtazapine suppresses Con A-mediated upregulation of hepatic ICAM-1 expression. ICAM-1 immunohistochemistry of representative liver sections from vehicle, mirtazapine, Con A, and Con A + mirtazapine treated mice, showing: **(A,B)** limited sinusoidal expression and weak central vein endothelium expression of ICAM-1 in vehicle and mirtazapine treated mice. No ICAM-1 expression was detected in portal vein endothelium. **(B)** striking increase of hepatic ICAM-1 expression in sinusoidal endothelium and central veins at 16 h post-Con A treatment. **(C)** marked reduction in the Con A-induced increase in hepatic ICAM-1 expression post-mirtazapine treatment. Portal veins are indicated by a black arrow head, central veins by a double-headed arrow, and sinusoids by a single-headed arrow (100X).

As previously reported, the liver has low basal ICAM-1 expression (Figure 5A), which was unaffected by mirtazapine treatment (Figure 5B). Con A treatment strikingly increases hepatic ICAM-1 expression (22) (Figure 5C), and mirtazapine treatment significantly reduced the Con A-induced increase in hepatic ICAM-1 expression (Figure 5D).

### Mirtazapine Effects on Hepatic Serotonin and Histamine Levels

We were unable to detect histamine levels by ELISA in any of the treatment groups. Hepatic serotonin levels increased insignificantly in Con A treated vs. vehicle and mirtazapine treated mice; however, mirtazapine treatment in Con A treated mice significantly increased hepatic serotonin levels, compared to both control groups (hepatic serotonin levels [pg/mg protein]; vehicle: 127.2 ±17.2 vs. mirtazapine treated: 119.0 ± 12.2 vs. Con A: 157.0 ± 11.3 vs. Con A + mirtazapine: ^*^192.6 ± 23.1; ^*^p < 0.05 vs. vehicle and mirtazapine groups; *n* = 4–5 mice per group).

### Attenuation of Con A-Induced Production of TNFα, IL-6, and the Chemokine CXCL5 From Human Monocytes *in vitro*

**Figure 6:**
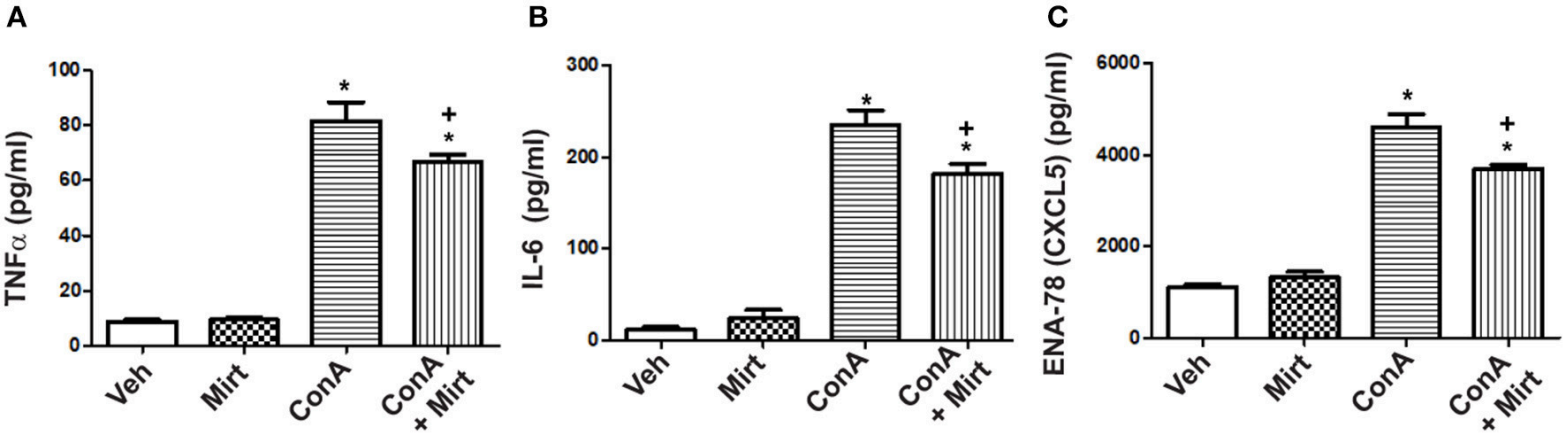
Mirtazapine attenuates TNFα, IL-6, and CXCL5 production by activated human monocytes/macrophages *in vitro*. Plastic adherent human CD14^+^ monocytes/macrophages were stimulated with Con A (5 μg/ml) for 24 h in the presence or absence of mirtazapine (10 μM). Levels of IL-6, TNFα, and CXCL5 were measured in culture supernatants using a Luminex^®^ assay. **(A–C)** Stimulation with Con A for 24 h induced a significant increase in culture supernatant levels of TNFα, IL-6, and CXCL5 (pg/ml). Mirtazapine treatment resulted in a significant attenuation of the Con A-induced release of all three mediators into culture supernatants. For all panels, ^*^p < 0.0001 Con A + vehicle and Con A + mirtazapine vs. vehicle + DMSO and mirtazapine treated control groups. **(A)** ^+^*p* < 0.05 for Con A + vehicle vs. Con A + mirtazapine group. **(B,C)** ^+^*p* < 0.01 Con A + vehicle vs. Con A + mirtazapine group (*n* = 4 for all groups).

To determine whether mirtazapine directly alters activation-induced inflammatory mediator production by human monocytes/macrophages, primary human CD14^+^ cells were isolated and treated *in vitro* with Con A, in presence or absence of mirtazapine. Incubation of healthy donor CD14^+^ monocytes with Con A *in vitro* resulted in a significant increase in culture supernatant levels of TNFα, IL-6, and the neutrophil-relevant chemokine CXCL5 (Figures 6A–C). Mirtazapine treatment significantly attenuated this Con A-induced increased production of these three mediators (Figures 6A–C).

### Impact of Mirtazapine Treatment on Con A-Induced Hepatic CD4 T Cell Recruitment and Activation, and on Human CD4 T Cell Production of Cytokines *in vitro*

**Figure 7:**
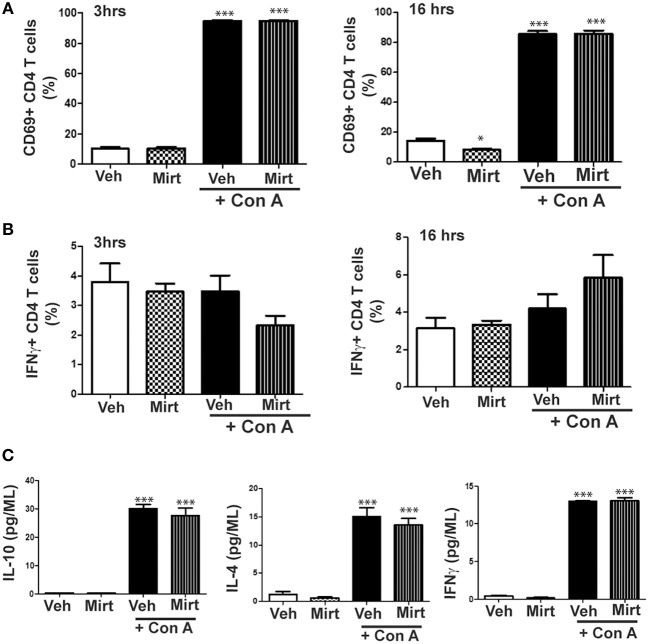
Mirtazapine does not alter Con A-induced mouse CD4 T cell activation *in vivo* or human CD4 T cell cytokine production *in vitro*. **(A)** Con A treatment significantly increases hepatic CD4 T cell expression of the cellular activation marker CD69 at 3 and 16 h post-Con A treatment, which was not altered by mirtazapine (20 mg/kg) treatment. ^***^p < 0.001 vs. vehicle and mirtazapine treated groups. Mirtazapine significantly reduced CD69 expression on CD4 T cells at 16 h post-treatment compared to vehicle treated mice. ^*^p < 0.05 vs. vehicle treated group (*n* = 5 mice/group). **(B)** Hepatic CD4 T cell production of IFNγ at 3 or 16 h post-vehicle, mirtazapine, Con A and Con A + mirtazapine treatment was not significantly different in all treatment groups (*n* = 5 mice/group). **(C)** Freshly isolated human peripheral blood CD4 T cells were stimulated with Con A (5 μg/ml) *in vitro* for 24 h in the presence or absence of mirtazapine (10 μM) and supernatant levels of IFNγ, IL-10, and IL-4 measured by Luminex. Con A stimulation significantly increased production of all three cytokines compared to vehicle and mirtazapine alone treated groups, and mirtazapine did not alter Con A stimulated secretion of any of the three cytokines. ^***^p < 0.001 vs. vehicle and mirtazapine alone groups (*n* = 3–4 for all groups).

Mirtazapine treatment alone did not alter hepatic CD4 T cell numbers at 3 or 16 h, but mirtazapine treatment significantly augmented the recruitment of CD4 T cells to the liver 16 h post-Con A treatment (number of CD4 T cells/liver (x 10^4^); vehicle: 6.21 ± 0.79 [SEM] vs. mirtazapine: 4.11 ± 0.38 vs. Con A: ^*^33.07 ± 3.86 vs. Con A + mirtazapine: ^*^#48.81 ± 6.49; *n* = 4–5 mice/group; ^*^p < 0.001 vs. control groups; ^#^*p* < 0.01 vs. Con A alone group). Furthermore, Con A treatment induced a significant ~8-fold increase in the proportion of CD69 expressing hepatic CD4 T cells which was of similar magnitude in Con A-treated mice that did or did not receive mirtazapine (Figure 7A). Con A treatment did not significantly increase the proportion of CD4 T cells expressing IFNγ, and mirtazapine did not alter either the basal or Con A stimulated proportion of IFNγ producing cells (Figure 7B). In cultured human CD4 T cells Con A treatment significantly enhanced production of the cytokines IL-10, IL-4, and IFNγ, which was unaltered by mirtazapine treatment (Figure 7C).

## Discussion

The aims of this study were to determine the impact of the atypical antidepressant mirtazapine on hepatic innate immune responses that have been previously shown to critically regulate the development of Con A-induced immune-mediated liver injury; a model of human AIH (19, 20, 26). Our data reveal that mirtazapine treatment dose-dependently reduces Con A-mediated liver damage and significantly attenuates Con A-mediated activation of hepatic innate immune responses. Specifically, mirtazapine treatment attenuated Con A-driven activation of hepatic macrophages/monocytes and significantly reduced Con A-induced increases in hepatic expression of the cytokines TNFα and IL-6, and the chemokines CXCL1 and CXCL2, and hepatic expression of the neutrophil adhesion molecule ICAM-1. Importantly, these cytokines, chemokines and adhesion molecule play key roles in the recruitment of neutrophils into the liver during the development of Con A-mediated liver injury (19, 21–25, 43). Moreover, these mirtazapine-related effects in Con A hepatitis were associated with a significant reduction in the hepatic recruitment of neutrophils associated with Con A treatment. In contrast, mirtazapine was without effect in suppressing liver injury in the αGalCer model of hepatitis which does not require hepatic macrophage/monocyte activation for induction of liver injury (27). Consistent with our findings in the Con A model, we showed that mirtazapine also suppresses the LPS-stimulated release of cytokines and chemokines from human CD14^+^ monocytes, but not CD4 T cells, *in vitro*. Our data reveal that mirtazapine has a profound suppressive impact on the activation of hepatic innate immune processes, which in turn significantly impairs the development of immune-mediated liver injury.

Historically, adaptive immune processes have been implicated as the main driver of autoimmune disease, with a limited role assigned to innate immune responses (1). However, more recent experimental and clinical evidence has indicated that innate immunity plays a key role as an early driver of later adaptive immune responses, and the ultimate development of autoimmune disease (2, 3, 49). Similarly, accumulating clinical and experimental evidence suggests that a parallel hierarchal process may also exist in AIH (5–8, 21, 24, 25). Our current findings are consistent with previous reports indicating that activation of hepatic innate immune responses, including hepatic macrophage/monocyte activation, and associated neutrophil recruitment to the liver, occurs early during the development of Con A hepatitis. However, we now show that mirtazapine, a widely used atypical antidepressant, attenuates Con A-induced activation of the hepatic innate immune cascade. Specifically, mirtazapine treatment significantly attenuated Con A-mediated activation of hepatic macrophages/ monocytes and associated neutrophil recruitment-relevant liver cytokine and chemokine expression, as well as the upregulation of the important neutrophil adhesion molecule ICAM-1 within the liver. These mirtazapine-induced changes within the liver were associated with a significant reduction in the hepatic infiltration of neutrophils associated with a striking histological and biochemical reduction in Con A-mediated liver damage. In contrast, we found that selective inhibition of single mirtazapine-relevant serotonin or histamine receptors individually did not attenuate Con A hepatitis severity (and in fact, in some cases actually worsened Con A hepatitis) (Supplementary Figure 1). These findings suggest that the *combination* of serotonergic and/or histamine receptors that are impacted by mirtazapine (13–15), are important regulators of innate immunity in the liver. In this study we used the Con A model of autoimmune hepatitis in which adaptive immune responses critically regulate the development of liver injury (44). Moreover, adaptive immunity is also implicated clinically in the development of autoimmune hepatitis in patients (45). Therefore, although we did not observe a significant impact of mirtazapine on early hepatic adaptive T cell immune activation, it is possible that mirtazapine may impact later adaptive immune activation with associated alterations in the clinical expression of liver autoimmunity.

Mirtazapine is a tetracyclic molecule that exhibits a complex pharmacology, having both central and peripheral effects (13, 30), acting as a 5HT_2A_/5HT_2B_ receptor antagonist, 5HT_2C_ receptor inverse agonist, and an antagonist for 5HT_3_ and histamine (H_1_) receptors (12, 50). Given its' broad range of receptor activity and excellent safety profile, mirtazapine has been increasingly used clinically to treat a broad range of symptoms including depression, anxiety, anorexia, insomnia, and nausea/vomiting (12). However, in addition to its effects on symptoms, there is evidence that mirtazapine also impacts systemic immunity (11, 51–53). Moreover, in a number of preclinical disease models inhibition of many of the individual receptor subtypes blocked by mirtazapine can also modulate inflammatory responses (15, 16, 54). Importantly, all of the receptor subtypes impacted by mirtazapine treatment (except α2 adrenergic receptors) can be expressed on macrophages/monocytes, and differentially regulate cellular activation, migration and cytokine/chemokine release (14, 16, 17, 55–57). Given that mirtazapine likely impacts signaling in immune cells by acting through a combination of these numerous receptor subtypes, it is reasonable to speculate that mirtazapine may exhibit unique inflammation-modulating effects compared to inhibition of individual receptors, possibly by modulating macrophage/monocyte responses in tissues. It is widely recognized that the endogenous ligands for these receptors, serotonin and histamine, can significantly modulate immunity (14–16, 54). Moreover, hepatic levels of serotonin and histamine are increased in many liver diseases (58–60). Furthermore, using an administrative dataset from a large British primary care database of 11.1 million patients, we found a striking reduction in adverse liver outcomes, including hepatic decompensation, transplantation, and mortality, in patients treated with mirtazapine suffering with the autoimmune liver disease primary biliary cholangitis (18). Our current findings in the Con A model of autoimmune liver injury are consistent with these clinical observations, and suggest that attenuation of hepatic innate immune responses by mirtazapine, mediated by effects on liver macrophage/monocyte activation and neutrophil recruitment, may also potentially contribute to improved clinical outcomes in patients with a number of autoimmune liver diseases.

TNFα is a critical regulator of autoimmunity, including AIH in both patients and animal models (19, 22, 41). In the Con A model of autoimmune hepatitis, hepatic macrophages/monocytes are the main TNFα producing cell type, and TNFα released by these cells drives downstream innate and adaptive immune responses within the liver (21, 22, 40, 61). Our current data shows that mirtazapine treatment suppresses Con A-induced hepatic macrophage/monocyte TNFα production, and significantly attenuates the Con A-induced increase in hepatic TNFα levels, which in turn inhibits the development of Con A hepatitis. Interestingly, mirtazapine treatment alone resulted in a small but significant increase in hepatic TNFα levels; a finding suggesting that mirtazapine treatment may activate hepatic macrophages. Consistent with our current findings, treatment of patients with mirtazapine can also cause an increase in plasma TNFα levels, suggesting a similar impact of mirtazapine on macrophages may occur in humans (62).

CD80 is expressed on antigen presenting cells, including macrophages, and plays an important role as a costimulatory signal for T cell activation (63). KCs express very low levels of CD80 (64). MHC II is also expressed on tissue macrophages and plays a critical role in immunity by presenting extracellular antigen to activate T cells (65). Moreover, both CD80 and MHC II expression can be induced on antigen presenting cells by IFNγ (66). We found that mirtazapine treatment differentially altered both CD80 and MHC II expression on hepatic macrophage/monocyte subtypes. However, overall the mirtazapine-related effects on macrophage/monocyte activation with respect to CD80 and MHC II expression, were similar to its' effects on TNFα production; namely, suppression of Con A stimulated expression of both CD80 and MHC II. These observations indicate that mirtazapine has an immunomodulatory role in monocyte/macrophage activation during the development of immune mediated hepatitis, in a pattern consistent with the induction of a more anti-inflammatory or tolerogenic hepatic milieu.

In the Con A hepatitis model hepatic macrophages are the main cell type producing the chemokine CXCL2 (24, 67). Furthermore, CXCL2 plays a key role in the recruitment of neutrophils to the liver and in the ultimate development of Con A hepatitis (24). Consistent with its' impact on hepatic macrophage activation and TNFα production after Con A treatment, mirtazapine also suppressed Con A-induced increases in hepatic CXCL2 levels. These findings suggest that mirtazapine-related effects on hepatic macrophages play a key role in its' regulatory effect in autoimmune liver disease.

In summary, our data reveal that the antidepressant mirtazapine exhibits robust immunomodulatory effects in suppressing immune-mediated liver injury. Our data suggest that mirtazapine mediates its' anti-inflammatory effects by interfering with the development of early innate immune responses within the liver; processes that have been postulated to be key regulators in the development of a variety of autoimmune diseases (2–4, 8, 49). Importantly, mirtazapine is widely available and has been used clinically to treat depression for over two decades, and is safe and generally well-tolerated (12, 13). Therefore, mirtazapine may represent a novel therapeutic approach for the treatment of autoimmune liver disease and warrants further study.

## Ethics Statement

All procedures were approved by the University of Calgary Animal Care Committee (protocol numbers AC14-0129, AC14-0128) and were performed in accordance with the guidelines of the Canadian Council on Animal Care.

## Author Contributions

WA and MGS performing all experiments and data analysis and interpretation. WA, AAS, KAS, and MGS participated in the study design and in the conceptual approach to experiments, as well as in critically appraising the data and in writing and editing the manuscript.

### Conflict of Interest Statement

The authors declare that the research was conducted in the absence of any commercial or financial relationships that could be construed as a potential conflict of interest.
