# Supplementary material for: The Antidepressant Mirtazapine Inhibits Hepatic Innate Immune Networks to Attenuate Immune-Mediated Liver Injury in Mice
Source: Front Immunol. 2019 Apr 12;10:803. doi: 10.3389/fimmu.2019.00803 (PMC6474187; doi:10.3389/fimmu.2019.00803)
Supplement: Supplementary file 1 [file Data_Sheet_1.docx]

| 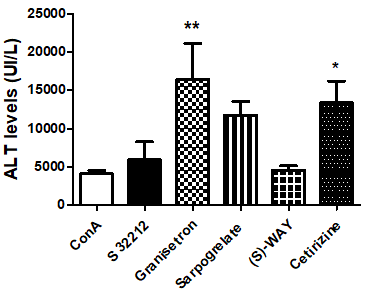 |
| --- |

**Supplementary Figure 1**. Administration of individual serotonin and histamine receptor antagonists do not attenuate Con A-induced hepatitis.

All antagonists were administered intraperitoneally except cetirizine dihydrochloride which was given via p.o. route (all 10 mg/kg). All mice received Con A and were treated with either vehicle or receptor antagonists individually. Receptor antagonists include: Sarpogrelate hydrochloride (selective 5-HT2a antagonist), granisetron hydrochloride (5-HT3 antagonist), S 32212 hydrochloride (5-HT2c inverse agonist; α2 antagonist), (S)-WAY 100135 dihydrochloride (selective 5-HT1a receptor antagonist). At 16 hrs post-Con A injection plasma samples were collected to evaluate liver injury as reflected by serum alanine aminotransferase (ALT) levels. **p<0.01 and *p<0.05 vs Con A group. N=4-5 mice per group
